# CHILDREN’S HEALTH: School Siting: EPA Says Location Matters

**DOI:** 10.1289/ehp.119-a19

**Published:** 2011-01

**Authors:** Bob Weinhold

**Affiliations:** **Bob Weinhold**, MA, has covered environmental health issues for numerous outlets since 1996. He is a member of the Society of Environmental Journalists

Fifty-three million U.S. children and 6 million employees spend much of the day in a public or private school.^1^ Pollution problems in these settings are so widespread that the Congress mandated in the Energy Independence and Security Act of 2007 that the U.S. Environmental Protection Agency (EPA) develop model guidelines for choosing healthier sites for new schools. On 17 November 2010, the agency released a draft of its new voluntary guidelines.^1^,^2^

About 1,900 new schools were built in the 2008–2009 school year, according to the EPA, continuing a relatively similar construction trend since 2002^3^ and bringing the total number of public and private schools to about 135,000.^1^ The number of existing schools in settings that could be harmful to children is unknown, says Peter Grevatt, director of the EPA Office of Children’s Health Protection.

The guidelines are designed mainly for use in siting new primary and secondary (K–12) schools, but the principles behind the guidelines could be adapted for many other existing and new settings where children spend long periods. They cover a wide range of topics, including toxicity on the school site and from nearby properties; other health-related issues such as bicycle and pedestrian access to increase student exercise; maximizing community use of the school; and minimizing disruption of relatively undisturbed environments.

Jason Hartke, vice president of national policy for the U.S. Green Building Council, is generally pleased with the congressional mandate and EPA’s actions so far. “There is a strong need for EPA guidelines,” he says. “This is another really important tool in the toolbox” for creating healthier schools.

Stephen Lester, science director for the Center for Health, Environment & Justice, also is generally supportive: “There’s a lot of good information in these guidelines.” But he says they offer too much wiggle room for allowing schools to be built on toxic sites, such as Superfund properties. He’d rather see language that sanctions such decisions only as a very last resort. That’s important, he says, because school districts “never have enough money for monitoring and maintenance,” even if the original planning, design, and engineering for mitigating toxicity problems were deemed acceptable. He also would prefer a no-exceptions guideline that directs use of the more-protective cleanup standard for residential use for all school sites.^4^

A broader concern is that many school districts may choose to ignore the voluntary guidelines. Interest in environmental health issues “is very spotty,” Lester says, especially when so many other issues—including site availability, zoning, and cost—are high priorities. Even in the U.S. Green Building Council’s LEED (Leadership in Energy and Environmental Design) voluntary certification process for schools,^5^ toxicity issues account for only 10 of the 110 optional points.^6^

The public can comment on the draft guidelines until 18 February 2011. A final version is scheduled for release in late 2011.
